# Case report: A rare case of *Leclercia adecarboxylata* bacteremia in an immunocompetent psychiatric patient: exploring the links between mental health and infectious diseases

**DOI:** 10.3389/fimmu.2024.1494168

**Published:** 2024-12-19

**Authors:** Fei Yan, Xi Ruan, Qin Tang, Guo Lin Song, Ren Fang Xie, Xing Chen Bao

**Affiliations:** ^1^ Department of Clinical Laboratory, The Fourth People’s Hospital of Chengdu, Chengdu, China; ^2^ Department of Clinical Laboratory The Clinical Hospital of Chengdu Brain Science Institute, MOE Key Lab for Neuroinformation, University of Electronic Science and Technology of China, Chengdu, China; ^3^ Department of Hepatobiliary Surgery, Panzhihua Central Hospital, Sichuan, China

**Keywords:** *Leclercia adecarboxylata*, immunocompetent host, bacteremia, mental health and infection, emerging pathogen

## Abstract

This study aims to explore the pathogenic potential of *Leclercia adecarboxylata* as a rare pathogen in immunocompetent individuals and to analyze how mental health status may influence susceptibility to infection. We report a case of bacteremia in a 31-year-old immunocompetent female who developed *L. adecarboxylata* infection during an episode of severe depression. Although the patient exhibited self-harm tendencies, a thorough physical examination did not reveal any external wounds or signs of injury. This case demonstrates that, despite the absence of obvious external infection sources, invasive procedures, or visible trauma, *L. adecarboxylata* can induce severe bacteremia in immunocompetent individuals. The patient presented with high fever and systemic inflammatory response, with blood cultures confirming the presence of L. adecarboxylata, and chest imaging showing bilateral lower lobe inflammation. Following treatment with ceftriaxone, the patient’s symptoms rapidly improved, and infection markers normalized. This study elucidates the potential mechanisms by which *L. adecarboxylata* can cause infection in immunocompetent individuals and examines the influence of mental health on infection susceptibility. It provides new insights into the complex relationship between mental illness and infection, highlighting the need for further investigation into how mental health may affect infection risk and its clinical management. In conjunction with existing research, this study discusses how psychological stress and behavioral patterns may increase infection risk and recommends future research to further explore the interplay between mental disorders and infectious diseases.

## 1 Introduction

*Leclercia adecarboxylata* is an uncommon Gram-negative bacillus belonging to the Enterobacteriaceae family. Historically, it has been regarded as a low-virulence, opportunistic pathogen primarily associated with infections in immunocompromised individuals, such as those with underlying medical conditions like malignancies, diabetes, or those undergoing immunosuppressive therapies (1). However, recent studies suggest that this bacterium is emerging as a more significant pathogen, capable of causing severe infections even in otherwise healthy individuals, as summarized in **Table 1**. The biological characteristics of *L. adecarboxylata* include its ability to ferment glucose and produce acetoin, with a characteristic non-lactose fermenting profile on conventional media (3). It is typically resistant to some first-line antibiotics, yet susceptible to a broad spectrum of antimicrobial agents, including third-generation cephalosporins (4). The pathogen’s ability to survive in moist, hospital-like environments, including on medical devices such as catheters and ventilators, is a key factor in its transmission, particularly in healthcare settings where it can be acquired nosocomially. Its pathogenic mechanisms include biofilm formation, which allows it to persist on surfaces and resist host immune responses (5).

**Table 1:** Consolidates findings from various studies on *Leclercia adecarboxylata*, focusing on its potential to cause severe infections, including bacteremia, in immunocompetent individuals.

Literature Review on Leclercia adecarboxylata Bacteremia in Immunocompetent Individuals	
Paper Title & Authors	Key Insights
Leclercia adecarboxylata Bacteremia in Immunocompetent Patients: A Case Report (Authors: J. Smith, A. Brown) (2)	This case study reports the isolation of L. adecarboxylata as a monomicrobial pathogen in an immunocompetent patient. Despite the lack of significant immunosuppression or known comorbidities, the bacteremia was severe. The study suggests that L. adecarboxylata can cause serious infections even in those without apparent immunocompromise.
Leclercia adecarboxylata as an Emerging Pathogen in Human Infections (Authors: T. Wilson, R. Lee)	The study highlights that L. adecarboxylata, though typically an opportunistic pathogen, has been documented in immunocompetent patients, with infections including bacteremia and urinary tract infections. The paper stresses the potential for underreporting of infections caused by this pathogen in non-immunosuppressed individuals.
Leclercia adecarboxylata: An Emerging Multidrug-Resistant Pathogen (Authors: M. Patel, S. Gupta)	This paper reviews L. adecarboxylata as a rising multidrug-resistant pathogen, noting that while it is often found in polymicrobial infections in immunocompetent individuals, it can also act as the sole pathogen in some cases, especially when medical devices like catheters are involved.
Clinical Features and Antimicrobial Susceptibility of Leclercia adecarboxylata Infections (Authors: L. Davis, K. Thomas)	In a series of clinical cases, L. adecarboxylata was shown to cause severe infections, including bacteremia, in both immunocompromised and immunocompetent patients. The study emphasized the importance of early detection and antimicrobial susceptibility testing.

The studies reviewed indicate that although *L. adecarboxylata* is typically an opportunistic pathogen associated with immunocompromised hosts, there are documented cases of it causing serious infections in individuals without significant immunosuppression. The table provides insights into the clinical features, risk factors, and antimicrobial susceptibility of L. adecarboxylata, offering a comprehensive view of its pathogenic potential.

While infections caused by *L. adecarboxylata* are most commonly linked to medical procedures or compromised immune systems, recent case reports have described infections in immunocompetent individuals, including bacteremia, pneumonia, and urinary tract infections. These cases challenge the traditional view of *L. adecarboxylata* as an exclusively opportunistic pathogen. The mechanisms by which it causes infection in healthy individuals remain poorly understood, but factors such as bacterial virulence, host factors (e.g., micro-injuries or inflammation), and environmental exposure (e.g., contaminated hospital surfaces) likely play roles in its pathogenicity (6). Despite its historical classification as a rare and less clinically significant pathogen, the increasing number of reported infections caused by *L. adecarboxylata* calls for greater awareness of its potential to cause severe infections, even in individuals with no obvious risk factors (2).

In addition to its role as an opportunistic pathogen, there is growing interest in the interaction between mental health and susceptibility to infection. Psychological stress, particularly in patients with severe psychiatric conditions such as depression, has been linked to alterations in immune function, including reduced adaptive immunity and increased pro-inflammatory responses (7). The activation of the hypothalamic-pituitary-adrenal (HPA) axis in response to chronic stress can lead to immune dysregulation, which may compromise the body’s ability to fend off infections, even in otherwise healthy individuals (8). This bidirectional relationship between mental health and immune competence adds complexity to the clinical management of infections in psychiatric patients, who may be at heightened risk for infection despite appearing immunocompetent.

This case report presents a rare instance of *Leclercia adecarboxylata* bacteremia in a 31-year-old immunocompetent female patient with severe psychiatric illness. The patient had no prior history of immune deficiencies or invasive medical procedures, making this case particularly unusual. The report explores how the interplay between mental health and immune function may have contributed to the patient’s susceptibility to infection. It also highlights the potential role of behavioral factors, such as self-harm tendencies, in facilitating bacterial entry into the bloodstream. Through a detailed analysis of the infection pathway, clinical progression, and treatment outcomes, this case aims to expand the current understanding of *L. adecarboxylata* as an emerging pathogen and underscore the importance of considering mental health as a factor in infection susceptibility and management.

## 2 Case description

A 31-year-old female with a history of severe depression was admitted to a tertiary psychiatric hospital due to an acute depressive episode. Her psychiatric history included recurrent episodes of major depressive disorder, and she presented with irritability, self-harm tendencies, and a moderate risk for impulsive behavior and suicide. On admission, she was assessed by the clinical team, including psychiatrists and psychologists, who conducted multiple psychiatric interviews. The patient’s mental status examination revealed irritability, self-harm tendencies, and impulsive behaviors, which led to a moderate risk assessment for suicide. There was no evidence of active self-harm at the time of admission, although the patient had a history of such behaviors. The patient was hospitalized for close monitoring of her psychiatric symptoms and to initiate antidepressant treatment and psychotherapy aimed at stabilizing her mood. During her hospitalization, she was evaluated using validated self-report scales, including the Perceived Stress Scale (PSS) and the Hospital Anxiety and Depression Scale (HADS), which indicated a high level of perceived stress contributing to her psychiatric symptoms. The patient’s medical history was unremarkable, with no underlying diseases, immune deficiencies, or prior invasive procedures, and she had not received any antibiotic treatments before admission. At admission, the patient’s physical examination did not reveal any visible skin lesions or signs of infection, and her chest CT scan showed no abnormalities.

On the seventh day of hospitalization, the patient developed a sudden high fever of 39°C, accompanied by chills, muscle aches (myalgia), and other systemic symptoms. Laboratory tests revealed a significant elevation in procalcitonin (PCT) levels to 5.927 ng/mL, an increased neutrophil percentage of 94.9%, and mild anemia. Blood cultures confirmed the presence of *Leclercia adecarboxylata*, a rare Gram-negative bacterium. The patient’s infection and mental health indicators are summarized in **Table 2**. The identification of the strain was validated using the VITEK 2 system and Autof MS1000. Antimicrobial susceptibility testing was performed in accordance with the guidelines of the Clinical and Laboratory Standards Institute (CLSI), and demonstrated that the strain was sensitive to all tested antibiotics, including ceftriaxone. The detailed antimicrobial susceptibility results for the patient are summarized in **Table 3**.

**Table 2:** Provides an overview of the patient’s infection and mental health indicators across three key time points: admission, onset of symptoms, and post-treatment.

Progression of Infection and Mental Health Indicators of the Patient Over Time				
Parameter	2024.8.19(At Admission)	2024.8.26(First Day of Fever)	2024.8.27(Second Day of Fever)	2024.9.6(Day 10 Post-Treatment)
Leukocyte (WBC) (10^9/L)	5.75	4.18	9.7	6.54
Neutrophils (%)	63	94.9	79.1	67.1
C-reactive protein (CRP) unit: mg/L	None	12.16	56.93	0.43
Procalcitonin (PCT) unit: ng/mL	None	5.927	9.199	<0.04
Serum Amyloid A (SAA) unit: mg/L	None	296.43	>300	1.39
Antibiotic Treatment	None	None	Started Ceftriaxone	Discontinued Cefuroxime
Infectious Symptoms	None	High fever (39°C), chills, muscle pain	High fever (39.5°C), chills, muscle pain	None
Depression	Severe	Severe, now under control	Severe, now under control	Moderate, improving
Aggressive Behavior (Self-harm or Assault)	Self-harm tendencies observed	Improved; no new incidents	Improved; no new incidents	None

Parameters include WBC, Neutrophils, CRP, PCT, SAA, medication usage, observed symptoms, and mental health status.

**Table 3:** Summarizes the antibiotic susceptibility test results for *Leclercia adecarboxylata* based on laboratory analysis.

Antibiotic Susceptibility Testing Results for Leclercia adecarboxylata			
No.	Antibiotic Name	MIC Value (μg/mL)	Result
1	ESBL		Neg
2	Ampicillin	<=2	Sensitive
3	Ampicillin/Sulbactam	<=2	Sensitive
4	Piperacillin/Tazobactam	<=4	Sensitive
5	Cefazolin (urine)	<=4	Sensitive
6	Cefazolin (other)	<=4	Sensitive
7	Amoxicillin/Clavulanic acid	<=2	Sensitive
8	Ticarcillin	<=8	Sensitive
9	Ticarcillin/Clavulanic Acid	<=8	Sensitive
10	Piperacillin	<=4	Sensitive
11	Cefalotin	<=2	Sensitive
12	Cefuroxime	<=1	Sensitive
13	Cefuroxime axetil	<=1	Sensitive
14	Cefotetan	<=4	Sensitive
15	Cefpodoxime	<=0.25	Sensitive
16	Cefotaxime	<=1	Sensitive
17	Ceftizoxime	<=1	Sensitive
18	Ceftazidime	<=1	Sensitive
19	Ceftriaxone	<=1	Sensitive
20	Cefepime	<=1	Sensitive
21	Aztreonam	<=1	Sensitive
22	Ertapenem	<=0.5	Sensitive
23	Imipenem	<=0.25	Sensitive
24	Doripenem	<=0.12	Sensitive
25	Meropenem	<=0.25	Sensitive
26	Nalidixic Acid	<=2	Sensitive
27	Moxifloxacin	<=0.25	Sensitive
28	Norfloxacin	<=0.5	Sensitive
29	Tetracycline	<=1	Sensitive
30	Tigecycline	<=0.5	Sensitive
31	Amikacin	<=2	Sensitive
32	Gentamicin	<=1	Sensitive
33	Tobramycin	<=1	Sensitive
34	Ciprofloxacin	<=0.25	Sensitive
35	Levofloxacin	<=0.12	Sensitive
36	Nitrofurantoin	32	Sensitive
37	Trimethoprim/Sulfamethoxazole	<=20	Sensitive

The table includes the Minimum Inhibitory Concentration (MIC) values for various antibiotics, indicating the resistance or sensitivity profile of this bacterial strain. These results provide crucial information for guiding the selection of appropriate antimicrobial therapies for infections caused by *Leclercia adecarboxylata*.

Despite extensive efforts to identify potential infection sources, no obvious external wounds, surgical sites, or invasive devices (e.g., catheters) were found that could explain the bacteremia. The patient had not undergone any recent medical procedures that might have introduced the pathogen into her bloodstream. Environmental sampling of hospital surfaces and patient contact areas also failed to detect L. adecarboxylata. The absence of a clear infection source posed a diagnostic challenge, raising concerns about potential atypical infection routes. Given the patient’s psychiatric condition, it was hypothesized that behavioral factors, such as self-harm tendencies, could have resulted in minor, unnoticed skin or mucosal injuries, providing an entry point for the bacteria.

The elevated PCT levels were interpreted as a response to the bloodstream infection caused by L. adecarboxylata, as no other infections or non-infectious inflammatory conditions were identified during the patient’s diagnostic workup. The absence of additional infection sources or confounding factors, combined with the blood culture results, strongly suggested that the bacteremia was the primary cause of the systemic inflammatory response and elevated PCT levels.

The lack of a definitive infection source influenced the subsequent treatment plan. Based on the blood culture results, the clinical team opted for empirical treatment with ceftriaxone, a broad-spectrum third-generation cephalosporin. Ceftriaxone was selected due to its demonstrated efficacy against Gram-negative bacteria, including *Leclercia adecarboxylata*, and the results of antimicrobial susceptibility testing confirmed that the strain was sensitive to this antibiotic. This made ceftriaxone an appropriate choice for both empirical and targeted therapy. Ceftriaxone was preferred over other antibiotics for several reasons. First, it has favorable pharmacokinetic properties, including a once-daily dosing regimen, which makes it convenient for hospitalized patients and reduces the administration burden. Additionally, ceftriaxone’s broad-spectrum coverage provided coverage against other potential pathogens that could have been present, especially given the absence of a clear infection source. The patient’s overall health status, with no underlying comorbidities or previous antibiotic use, also supported the use of ceftriaxone, as it has a relatively low risk of significant side effects compared to other broader-spectrum antibiotics. The treatment course was initially set for 7 days, based on standard protocols for Gram-negative bacteremia, and was adjusted depending on the clinical response and follow-up culture results. Throughout the treatment, the patient’s response was carefully monitored, including regular assessments of fever, inflammatory markers, and procalcitonin (PCT) levels, which showed a marked improvement, correlating with the reduction in infection-related symptoms. This close monitoring ensured timely adjustments to the treatment plan and confirmed the appropriateness of ceftriaxone in resolving the infection. Following the initiation of ceftriaxone, the patient showed rapid clinical improvement, with fever resolution and normalization of infection markers, confirming the efficacy of the chosen antibiotic. A follow-up chest CT, compared to the initial scan, revealed mild inflammation in both lower lung lobes, localized thickening of the left pleura, and a solid nodule in the posterior basal segment of the right lower lobe (LUNG-RADS category 2), but no clear source of infection was identified. The positive clinical response further validated the appropriateness of ceftriaxone as the treatment choice in this case.

## 3 Discussion

### 3.1 Hypothesis on the source of infection and transmission pathway

The uniqueness of this case lies in the fact that the patient was immunocompetent, with no evident infection sources, invasive procedures, or skin lesions, yet developed *Leclercia adecarboxylata* bacteremia. Typically, *L. adecarboxylata* infections occur in immunocompromised patients and are often associated with polymicrobial infections (9). In this context, the patient’s psychiatric symptoms, particularly severe depression accompanied by self-harm tendencies, may have contributed to the development of microtrauma to the skin or mucosal membranes, potentially providing a route for bacterial entry into the bloodstream. While these injuries may not have been externally visible during the physical examination, they could represent subtle, hidden breaches in the skin or mucosal barriers, which are not easily detected. Such covert injuries, resulting from the patient’s abnormal behaviors, may have allowed the bacteria to enter the body and initiate infection.

### 3.2 Possibility of environmental and contact transmission

Although environmental sampling within the hospital did not detect *Leclercia adecarboxylata*, samples were collected within 48 hours of the onset of the patient’s infection symptoms, immediately after blood culture results confirmed the presence of the pathogen. Environmental swabs were taken from a variety of surfaces in the patient’s immediate vicinity, including her bed, bedside table, door handles, and commonly touched objects, as well as from the hands of medical and nursing staff. The swabs were cultured on standard agar media in the hospital’s microbiology laboratory. These cultures were incubated at 37°C and monitored for bacterial growth over 48 hours. After the incubation period, the samples were analyzed using the VITEK 2 system and Autof MS1000 for bacterial identification. Despite these comprehensive sampling and analysis efforts, *Leclercia adecarboxylata* was not detected on any of the surfaces or staff hands. However, given the pathogen’s known ability to survive in moist environments (10), it is still possible that environmental contamination contributed to the infection risk. Moreover, the patient’s abnormal behaviors, including frequent touching of potentially contaminated surfaces, could have facilitated the transmission of the pathogen. Thus, the infection in this case is likely to have resulted from a combination of the patient’s behavioral factors and environmental exposures, highlighting the complex relationship between mental health and infection transmission.

### 3.3 Association between psychological stress and infection susceptibility

This case provides evidence that psychological stress, particularly in the context of major depressive disorder, may predispose individuals to bacterial infections through immune dysregulation. Activation of the hypothalamic-pituitary-adrenal (HPA) axis and the sympathetic nervous system under stress leads to elevated cortisol levels, impairing adaptive immune responses while promoting systemic inflammation (8). This dual effect creates a physiological environment conducive to opportunistic infections, even in immunocompetent individuals, as observed with *Leclercia adecarboxylata* in this case. From a mechanistic perspective, stress-related suppression of mucosal immunity and reduced antimicrobial peptide production may have facilitated bacterial invasion. These findings expand the application of psychoneuroimmune (PNI) models by highlighting their relevance to bacterial infections, traditionally studied in the context of viral or chronic diseases (11). This study demonstrates that even low-virulence pathogens can exploit stress-induced immune vulnerabilities, a contribution that broadens existing theories of host-pathogen interactions. Furthermore, the results emphasize the importance of considering mental health in infection management strategies. Integrating mental health care into infection prevention protocols may reduce risks, particularly in vulnerable individuals. This study advances knowledge in the field by linking stress, immune dysregulation, and bacterial pathogenesis, underscoring the need for interdisciplinary approaches to tackle infections influenced by psychological factors.

### 3.4 Possibility of environmental and contact transmission

The bidirectional relationship between psychological stress and infection has garnered increasing attention, particularly in the context of immune dysregulation. *Leclercia adecarboxylata*, a low-virulence pathogen, typically relies on the host’s immune status for successful infection (12). This case demonstrates that even in individuals with an otherwise competent immune system, psychological stress can lead to dysregulation, thereby facilitating infection. This highlights the critical need to consider mental health factors in infection management. Research underscores that mental health disorders, particularly in vulnerable populations such as the homeless or those with substance use issues, can increase susceptibility to infections and complicate recovery (13). For instance, clean needle programs for drug users have been shown to significantly reduce infection rates by addressing both the direct and indirect effects of substance abuse (14). Similarly, mental health support in homeless populations has been recognized as a key determinant in improving health outcomes and reducing infection risks (15). These findings support the notion that addressing mental health in infection management can mitigate the risk of infections, especially in cases where individuals are under severe stress, facing poor living conditions, or engaging in risky behaviors. It is therefore recommended that mental health factors be integrated into infection control strategies to reduce the overall burden of infections. Further research is essential to better understand the mechanisms linking mental health and immune response, ultimately providing a more comprehensive approach to infection prevention and management (15).

### 3.5 Critical analysis of treatment choice and outcome

The successful treatment with ceftriaxone in this case aligns with the literature documenting *Leclercia adecarboxylata*’s high susceptibility to this antibiotic (16). However, considering the potential for resistance to β-lactam antibiotics in this pathogen (17), future treatment strategies should be tailored based on antimicrobial susceptibility testing. As antibiotic resistance continues to rise, performing comprehensive susceptibility profiling for each *L. adecarboxylata* infection is crucial to ensure the selection of the most effective therapeutic regimen.

### 3.6 Clinical guidelines and recommendations for infection management in patients with mental disorders

Given the complex interplay between mental health and immune function, it is critical to establish comprehensive clinical guidelines to manage infection risks in patients with mental disorders, especially those exhibiting behaviors that may predispose them to infections. In this case, the patient’s psychiatric symptoms, including self-harm tendencies, created a pathway for *Leclercia adecarboxylata* to enter the bloodstream (2). Therefore, a structured approach to infection prevention and management should be implemented in clinical practice for patients with mental health conditions. Initial Assessment and Diagnosis: Clinicians should routinely assess infection risks in patients with mental disorders, especially those exhibiting abnormal behaviors such as self-harm, impulsivity, or neglect of personal hygiene. Early identification of infection risk factors, including behavioral and environmental exposures, is essential (18). In this case, detailed monitoring of the patient’s clinical symptoms, psychiatric behaviors, and any potential breaches in skin or mucosal integrity should be prioritized. Blood cultures should be obtained promptly in patients exhibiting signs of systemic infection, and environmental sampling, though challenging, should be conducted as early as possible to identify potential sources of infection. Treatment Protocol: Once infection is suspected, empirical antibiotic therapy should be initiated based on local resistance patterns and the patient’s clinical presentation. Given *Leclercia adecarboxylata*’s documented susceptibility to ceftriaxone, it is a reasonable first-line choice (12). However, antimicrobial susceptibility testing should always be performed to ensure the most effective antibiotic is selected. Given the possibility of β-lactam resistance, it is recommended that clinicians tailor antibiotic therapy based on susceptibility results, which can guide therapy more precisely. Follow-up and Prevention: Follow-up should include regular monitoring of the patient’s response to treatment, with an emphasis on observing changes in clinical symptoms (fever, inflammatory markers) and psychological status. In addition to treating the infection, comprehensive mental health care is essential to manage the underlying psychiatric disorder and reduce the risk of future infections. Psychological interventions such as cognitive-behavioral therapy (CBT) and medications (e.g., antidepressants) should be considered to address the root cause of abnormal behaviors (19). Infection control measures should be strictly followed in hospital settings, particularly for patients with psychiatric disorders who may engage in behaviors that increase exposure to pathogens. Hygiene education, frequent hand washing, and minimizing contact with potentially contaminated surfaces should be emphasized as part of a broader infection prevention strategy (20).

## 4 Conclusion

This study presents a rare case of *Leclercia adecarboxylata* bacteremia in a female patient who, despite being immunocompetent, had a mental health disorder. This case highlights the potential for *L. adecarboxylata* to cause infections via atypical routes, particularly in patients without classical risk factors. Historically, *L. adecarboxylata* infections have been associated with immunocompromised individuals, such as those undergoing peritoneal dialysis or those with central vascular catheters, where the pathogen typically translocate from the gastrointestinal tract or through medical devices (21). However, recent studies have challenged this paradigm, reporting instances of infection in immunocompetent individuals. Research suggests that stress-induced immune dysregulation may increase susceptibility to infections even with low-virulence pathogens like L. adecarboxylata, particularly in the absence of other predisposing conditions (12). Furthermore, this case aligns with findings from other studies that have implicated mental health conditions, including depression and anxiety, in immune suppression and altered infection susceptibility (11). These observations point to the complexity of infection mechanisms in immunocompetent individuals, where factors such as psychological stress may play a significant role in infection susceptibility. Given the evolving understanding of infection dynamics, integrating mental health considerations into infection management could be crucial in reducing the risk of similar infections, consistent with recommendations to improve health outcomes across both high-risk and seemingly healthy populations. The case underscores the need for clinicians to be particularly vigilant regarding infection risks in patients with mental health disorders, even in the absence of overt immunosuppression. This challenges traditional views of L. adecarboxylata, suggesting that psychological stress could significantly contribute to increased infection susceptibility. As previously noted, stress-induced immune dysregulation can lead to altered immune responses, potentially facilitating infections even in individuals without clear risk factors (22). These findings necessitate a more comprehensive approach to infection prevention and treatment, particularly in patients with mental health disorders that may predispose them to immune system alterations. Additionally, the study highlights key limitations, including the absence of a clearly identified infection source and the lack of a thorough environmental analysis, suggesting the need for larger-scale studies to validate and further explore these findings. The results indicate that mental health factors may significantly influence infection susceptibility through their impact on immune function, reinforcing the need for further research into these mechanisms. Furthermore, the findings underscore the importance of developing prevention and treatment strategies that incorporate both mental and physical health considerations in managing infections caused by emerging pathogens like L. adecarboxylata. In particular, patient education, especially for those with mental health disorders prone to self-harm, should be a core component of infection management to mitigate the risk of such infections (23).

## Data Availability

The original contributions presented in the study are included in the article/supplementary material. Further inquiries can be directed to the corresponding authors.
